# Discovery of small molecules that target a tertiary-structured RNA

**DOI:** 10.1073/pnas.2213117119

**Published:** 2022-11-21

**Authors:** Elena Menichelli, Bianca J. Lam, Yu Wang, Vivian S. Wang, Jennifer Shaffer, Katrina F. Tjhung, Badry Bursulaya, Truc Ngoc Nguyen, Todd Vo, Phillip B Alper, Christopher S McAllister, David H. Jones, Glen Spraggon, Pierre-Yves Michellys, John Joslin, Gerald F. Joyce, Jeff Rogers

**Affiliations:** ^a^Novartis Institutes for BioMedical Research, San Diego, CA 92121; ^b^Arrakis Therapeutics, Waltham, MA 02451; ^c^Velia Therapeutics, San Diego, CA 92130; ^d^Trotana Therapeutics, San Diego, CA 92121; ^e^Odyssey Therapeutics, Cambridge, MA 02142; ^f^The Salk Institute, La Jolla, CA 92037; ^g^Radial Therapeutics, Cambridge, MA 02142

**Keywords:** high-throughput screening, RNA aptamer, RNA–ligand interactions, RNA structure

## Abstract

There is growing interest in therapeutic intervention that targets disease-relevant RNAs using small molecules. While there have been some successes in RNA-targeted small-molecule discovery, a deeper understanding of structure–activity relationships in pursuing these targets has remained elusive. One of the best-studied tertiary-structured RNAs is the theophylline aptamer, which binds theophylline with high affinity and selectivity. Although not a drug target, this aptamer has had many applications, especially pertaining to genetic control circuits. Heretofore, no compound has been shown to bind the theophylline aptamer with greater affinity than theophylline itself. However, by carrying out a high-throughput screen of low-molecular-weight compounds, several unique hits were identified that are chemically distinct from theophylline and bind with up to 340-fold greater affinity. Multiple atomic-resolution X-ray crystal structures were determined to investigate the binding mode of theophylline and four of the best hits. These structures reveal both the rigidity of the theophylline aptamer binding pocket and the opportunity for other ligands to bind more tightly in this pocket by forming additional hydrogen-bonding interactions. These results give encouragement that the same approaches to drug discovery that have been applied so successfully to proteins can also be applied to RNAs.

## Significance

There have been increased efforts recently to “drug” RNA using orally bioavailable, low-molecular-weight compounds. This study combines two of the most powerful tools of modern drug discovery, high-throughput screening and structure-based design, to target the theophylline aptamer, a tertiary-structured RNA that binds theophylline with high affinity and selectivity. These efforts led to the discovery of compounds that bind the RNA with up to 340-fold greater affinity compared to theophylline. X-ray crystal structures were determined for the aptamer, both in isolation and in complex with various ligands, revealing the molecular basis for ligand binding. The results give encouragement that tertiary-structured RNAs are indeed druggable if one makes an effort similar to that used to address the most challenging protein targets.

RNA has long been a target of interest for small-molecule drug discovery. Some of the earliest work along these lines concerned the bacterial ribosome, which in retrospect proved to be the target of various antimicrobial agents, including aminoglycosides, chloramphenicols, and tetracyclines (1–4). Purposeful efforts to target structured RNAs with small molecules have also been successful, although with only limited examples thus far. A mass spectrometry-based screen of a large compound library led to the identification of compounds that bind and disrupt the internal ribosome entry site of hepatitis C viral RNA (5). Subsequent mechanistic and medicinal chemistry studies led to significantly improved versions of these compounds (6, 7). A recent study also employed a mass spectrometry-based screen, followed by hit expansion, to identify compounds that bind and disrupt the gene silencing domain within *Xist* RNA, thus inhibiting X-chromosome inactivation (8). Two independent studies used a high-throughput, cell-based screen to identify compounds that promote the inclusion of a skipped exon in *SMN2* pre-mRNA, thus restoring the expression of the SMN protein that is lacking in patients with spinal muscular atrophy (9, 10). In both cases, the identified compounds appear to modulate splicing by interacting with unique structural features of the RNA at the splice junction (10, 11), although those features are determined in part by protein components of the splicing complex. A different approach involves breaking down the target RNA into its secondary structural elements and, through a combination of computational and screening methods, seeks to identify ligands that bind those elements within the complete target RNA (12, 13).

Despite these successes, there are several reasons why RNA has generally been regarded as a more challenging drug target compared to protein. First, RNA tends to have a more flexible structure, especially when not bound by a protein partner. Second, the polyanionic backbone of RNA makes it difficult to form a deep hydrophobic pocket that would provide a favorable site for ligand binding and that instead attracts cationic ligands that tend to bind more superficially and promiscuously. Third, the simplicity of RNA’s primary and secondary structure, with only four subunits that organize into similarly shaped stem-loop elements, provides less opportunity to present unique features for molecular recognition. Yet, when one looks at tertiary-structured RNAs that bind small-molecule ligands, such as the many known aptamers and riboswitches (14, 15), it is clear that individual RNAs can have a unique structural personality.

In principle, it should be possible to invert the relationship between an aptamer and its cognate ligand: rather than starting with the ligand and evolving a corresponding aptamer, one might start with the structured RNA and conduct a high-throughput screen to discover corresponding small-molecule ligands. One example of this approach involved the bacterial riboswitch that senses flavin mononucleotide (FMN) and whose function is essential for the biosynthesis of riboflavin (16). A phenotypic screen of ~57,000 compounds was carried out to identify compounds that suppress bacterial growth in the absence of externally supplied riboflavin (17). This effort led to the discovery of a compound that occupies the FMN binding site of the riboswitch with an affinity only 13-fold weaker than FMN itself. The FMN riboswitch was not the only potential target for this phenotypic screen, but it proved to be an attractive one.

The present study purposively targeted the theophylline aptamer, a tertiary-structured RNA that has a special place in the hearts of RNA biochemists. Although obtained by in vitro selection long before the discovery of naturally occurring aptamers (18), it is the best studied of all aptamers. The NMR structure of the aptamer–theophylline complex was first determined 25 y ago (19). That structure revealed how multiple interdependent components of the RNA form a well-defined ligand binding pocket that enables theophylline to bind with submicromolar affinity while excluding closely related compounds such as caffeine (which differs by a single methyl group) by more than 10,000-fold. Until the present study, no X-ray crystal structure has been reported for the theophylline aptamer, nor has any ligand been found that binds to the aptamer more tightly than theophylline itself.

## Results

**Fig. 1.**
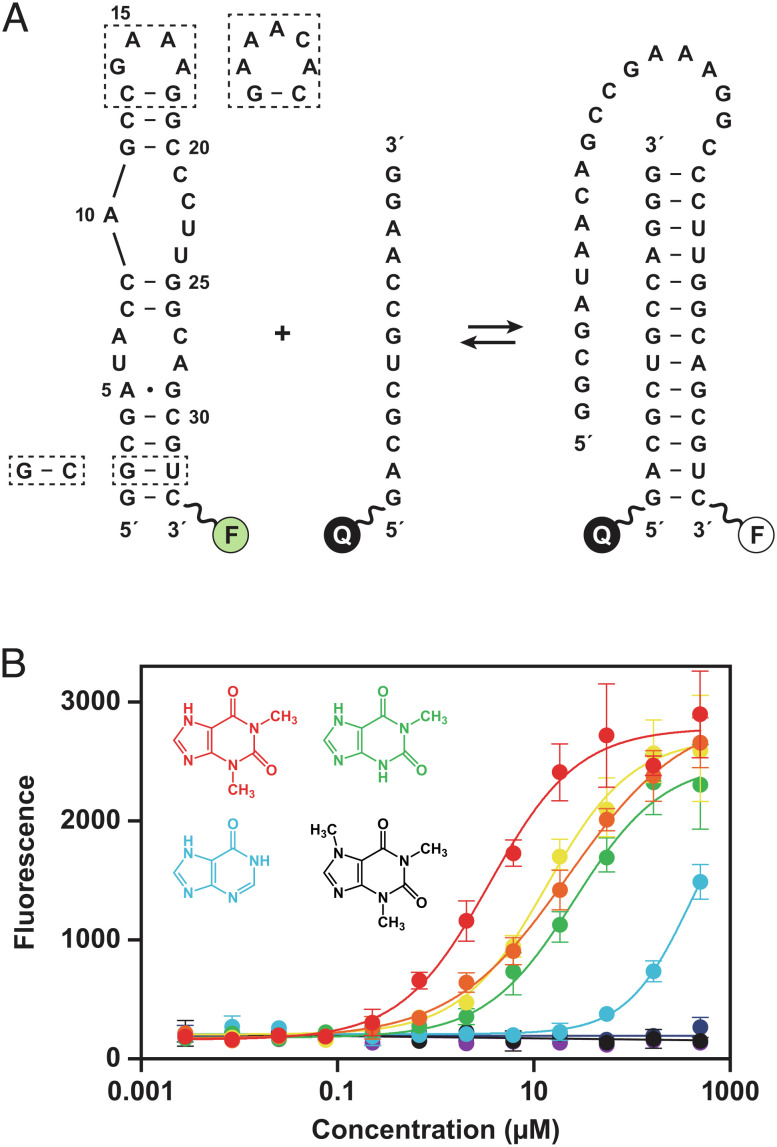
Strand invasion assay to screen for compounds that bind the theophylline aptamer. (*A*) Sequence and secondary structure of the theophylline aptamer, which was fluorescently labeled (F) at the 3′ end. Strand invasion by a partially complementary oligonucleotide, which has a fluorescence quencher (Q) at the 5′ end, results in decreased fluorescence. Two strategies were used to crystallize the aptamer, either replacing the GAAA tetraloop (nucleotides 14–17) with an AAACA pentaloop or replacing the G2-U32 wobble pair with a G–C Watson–Crick pair (dashed boxes). (*B*) Dose-dependent increase in fluorescence due to reduced propensity for strand invasion in the presence of theophylline (red), 1-carboxypropyl theophylline (orange), 3-methylxanthine (yellow), 1-methylxanthine (green), hypoxanthine (blue), 7-methylxanthine (indigo), 1,3-dimethyluric acid (violet), or caffeine (black). Values are based on four replicates, with error bars representing SE. The previously reported K_d_ values for binding of these compounds to the aptamer are 0.32, 0.93, 2.0, 9.0, 49, >500, >1,000, and 3,500 µM, respectively (18).

An assay was developed to conduct high-throughput screening of a library of ~1 million small molecules (molecular weight <600 Da) using strand invasion as a means to identify compounds that bind to the RNA aptamer. This is the same small-molecule library that has been used routinely to screen against various protein targets and cellular phenotypes as part of drug discovery campaigns. The strand invasion assay uses a fluorescently labeled aptamer together with a partially complementary oligonucleotide that carries a fluorescence quencher (Fig. 1*A* and *SI Appendix*, Table S1). When these two RNAs are separated, a fluorescent signal is detected, and this signal is diminished in proportion to the fraction of aptamers that have been invaded by the complementary oligonucleotide. The screen was carried out in the presence of each compound at 50 µM concentration, identifying those compounds that reduce the propensity for strand invasion and thus are likely to bind the aptamer. The assay was also run in the reverse mode by placing both the fluorescent dye and quencher on the aptamer, so that strand invasion resulted in increased fluorescence.

The strand invasion assay was validated by testing theophylline and seven theophylline analogs that have been evaluated previously in competitive binding experiments (18). Those prior experiments determined K_d_ values based on equilibrium dialysis, ranging from 0.32 µM for theophylline to 3,500 µM for caffeine. The strand invasion assay was performed in 12-point dose response with each of the eight compounds, which gave IC_50_ values that correspond well with the previously reported K_d_ values (Fig. 1*B*). However, these IC_50_ values should be regarded as only an estimate of K_d_ because they reflect the increase of fluorescence intensity as a function of concentration, which is only a surrogate of the binding event. Furthermore, the IC_50_ values are generally higher than the K_d_ for aptamer binding because inhibition of strand invasion is a more stringent requirement than simply binding to the target RNA. The stringency was optimized by testing invading strands containing various nucleotide analogs that bind more readily than unmodified RNA, settling upon 2′-deoxy,2′-fluoronucleotides at all 13 positions. Other compositions, including partial or complete substitution with locked nucleic acid, proved less efficacious with regard to the assay window.

The initial screen of 1,107,541 compounds resulted in 10,742 hits. These hits were counter-screened at 5 and 30 µM concentrations in four alternative assay formats to remove fluorescence artifacts and other false positives (see *Materials and Methods*). The resulting 153 confirmed hits (0.014% overall hit rate) have typical drug-like properties (20), with an average molecular weight of 290 Da, calculated octanol–water partition coefficient (cLogP) of 1.87, number of rotatable bonds of 3.3, and number of hydrogen-bond donors and acceptors of 1.9 and 5.0, respectively (*SI Appendix*, Fig. S1).

**Fig. 2.**
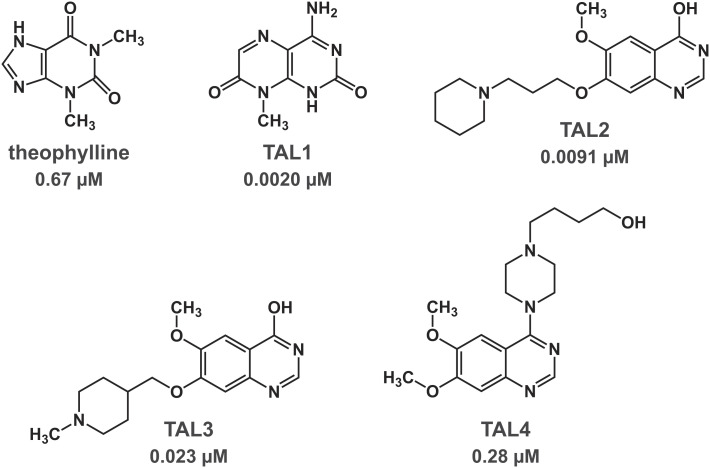
Chemical structure of theophylline and four compounds that were identified by high-throughput screening. The K_d_ is noted for binding of each compound to the theophylline aptamer, as determined by surface plasmon resonance in the presence of 150 mM NaCl and 5 mM MgCl_2_ at pH 7.4 and 25°C.

Each of the confirmed hits was evaluated in a dose–response assay to obtain IC_50_ values (*SI Appendix*, Fig. S2). Forty-six of these compounds exhibited an IC_50_ value lower than that of theophylline, including several examples with a chemical scaffold that is distinct from that of theophylline. Attention was focused on four of the hits, each with a 6,6 fused ring system, in contrast to the 6,5 fused ring system of theophylline (Fig. 2). One of the hit compounds, theophylline aptamer ligand 1 (TAL1), is an aminomethyl-substituted pteridinone that has a similar molecular weight as that of theophylline. The other three (TAL2, TAL3, and TAL4) have higher molecular weights, with differing substitutions of a common quinazolinone ring system.

The binding affinity of theophylline and each of the four hit compounds to the theophylline aptamer was determined by surface plasmon resonance. The K_d_ value for theophylline was 0.67 µM, which is in good agreement with the previously reported value (18). The K_d_ values for TAL1, TAL2, TAL3, and TAL4 were 0.0020, 0.0091, 0.023, and 0.28 µM, respectively (Fig. 2 and *SI Appendix*, Fig. S3 *A–E*). These values reflect a faster on-rate for all four compounds and a slower off-rate for all compounds except TAL4 compared to those of theophylline. The tightest binder is TAL1, with a K_d_ that is 340-fold lower than that of theophylline. There was no detectable binding of either theophylline or the hit compounds to a mutant form of the theophylline aptamer that contains a single U-to-C substitution at nucleotide position 24 (*SI Appendix*, Fig. S3 *F–H*).

In order to rationalize these findings and to better understand the chemical basis for molecular recognition of RNA, X-ray crystallography was used to determine the structure of the theophylline aptamer, both alone and in complex with theophylline and each of the four selected hits. Previous biophysical and modeling studies have shown that the aptamer undergoes a large conformational rearrangement upon binding theophylline (19, 21–23). Therefore, not surprisingly, initial crystallization trials of the ligand-free aptamer were unsuccessful. Two different strategies were used to obtain diffraction-quality crystals. The first involved replacing the GAAA tetraloop of the aptamer with an AAACA pentaloop, whereby the pentaloop structure is tightly bound by the antigen-binding fragment (Fab) of an antibody that is cocrystallized with the RNA (24, 25). The second strategy involved replacing the G2–U32 wobble pair at the base of the RNA aptamer by a G–C Watson–Crick pair, thus stabilizing the corresponding stem.

**Fig. 3.**
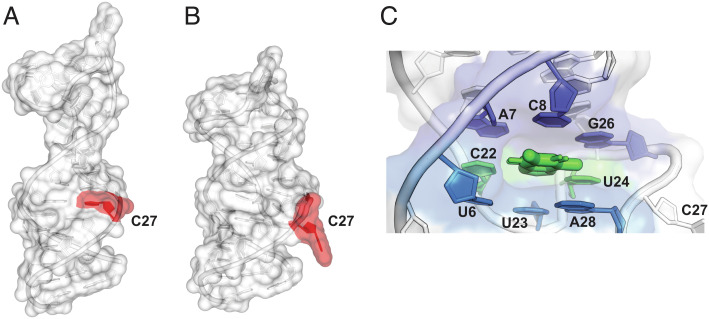
X-ray crystal structures of the theophylline aptamer, demonstrating conformational rearrangement upon binding of theophylline. (*A*) Structure of the ligand-free RNA within the Fab–RNA complex. (*B*) Structure of the ligand-bound form, shown in the same orientation. In the ligand-free structure, C27 (shaded red) is stacked between G26 and A28, whereas in the ligand-bound structure, C27 is flipped out of the helix to enable the formation of the S-turn. (*C*) Close-up view of the ligand-binding pocket (informed by the structure without the Fab), shaped by two internal loops brought into proximity by an S-turn formed by nucleotides C21–G29. Theophylline (thick green) is bound within the pocket as part of a base triple with C22 and U24 (green), which is stacked between two other base triples, one formed by A7–C8–G26 (indigo) and the other formed by U6–U23–A28 (blue).

Using the Fab cocrystallization strategy, the structure of the RNA aptamer in the absence of theophylline was solved at 1.81 Å resolution, using the Fab as the search model for molecular replacement (*SI Appendix*, Table S2). There were four copies of the complex in the asymmetric unit, with the majority of crystal contacts mediated by the surface of the Fab. The individual nucleotides could be localized, even though the electron density for the RNA was lower than that for the Fab. The RNA was found to exist in two conformations, each forming an extended hairpin, with most residues in a stacked arrangement and the notable absence of the theophylline binding pocket (Fig. 3*A*).

The apo-crystal was soaked with the aptamer ligand TAL2, and the structure of the complex was solved at 2.46 Å resolution (Fig. 3*B*). The electron density for the RNA remained lower than that for the Fab but was sufficient to reveal that a large conformational change had occurred upon ligand binding, forming the binding pocket. However, the ligand could not be precisely placed. Thus, to reveal atomic-level details of the RNA–ligand interaction, it was necessary to turn to the second crystallization strategy, with the stabilized aptamer stem and no protein component. This approach gave high-quality crystals (1.42–2.70 Å resolution) for the various aptamer–ligand complexes (Fig. 3*C*). The previous NMR structure of the theophylline-bound aptamer (19) was used to provide phasing information to solve the structure of these five complexes (26) (*SI Appendix*, Table S2 and Fig. S4).

**Fig. 4.**
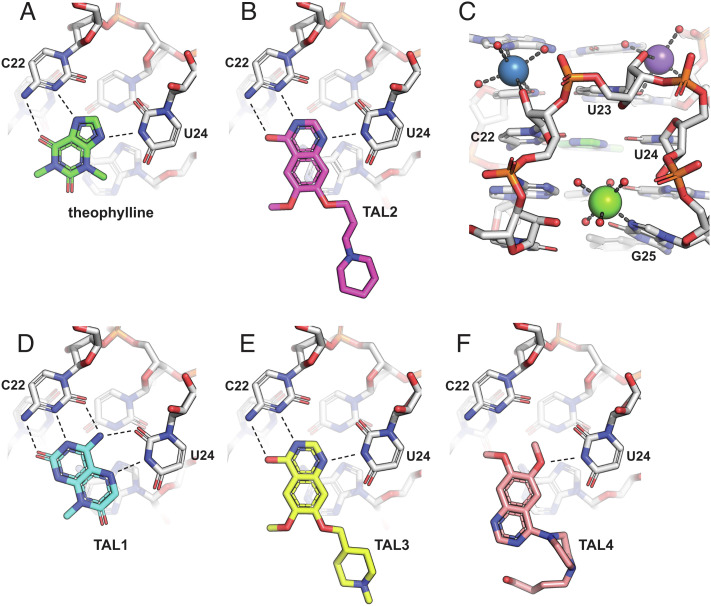
X-ray crystal structures of the theophylline aptamer with various bound ligands. (*A*) Theophylline forms a base triple with C22 (two hydrogen bonds) and U24 (one hydrogen bond), which stacks between two other base triples (A7–C8–G26 and U6–U23–A28). (*B*) TAL2 binds similarly to theophylline. (*C*) The binding pocket is stabilized by a Mg^2+^ ion (green sphere) that coordinates to N7 of G25, a Na^+^ ion (blue sphere) that coordinates to the 2′-hydroxyl of C22, and a second Na^+^ ion (purple sphere) that coordinates with U23 and forms water-mediated interactions with the sugar-phosphate of U24. Ion-coordinated water molecules are shown as red spheres. (*D*) TAL1 binds in the same pocket but with a third hydrogen bond to C22 and a second hydrogen bond to U24. (*E*) TAL3 binds similarly to TAL2. (*F*) TAL4 binds in the same pocket but with only a single hydrogen bond to U24.

The aptamer–TAL2 complex (without Fab) was present in two copies in the asymmetric unit. The structure revealed how the 4-hydroxyquinazoline of TAL2 nestles into the theophylline binding pocket, forming hydrogen-bonding interactions with both N3 and the exocyclic amine of C22 and with N3 of U24 (Fig. 4 *A* and *B*). The C22–TAL2–U24 triple stacks between the U6–U23–A28 and A7–C8–G26 base triples, just as seen with theophylline. A notable difference pertains to the methoxy and alkyl-piperidine groups of TAL2, which are exposed to the solvent and may account for the substantially enhanced affinity of this compound compared to theophylline. These substituents are not well-resolved in the crystal structure and do not appear to make direct contact with the aptamer but perhaps affect the entropic contribution to the free energy of binding, either via conformational dynamics or solvation. It is also possible that there are subtle differences in stacking energy when either theophylline or TAL2 are bound in the pocket.

The quinazolinone scaffold of the ligand occupies the theophylline binding site in a manner highly similar to the way theophylline is bound within the previously reported NMR structure (19) and in the crystal structure reported here. In both cases, C27 is flipped out of the extended hairpin, enabling residues C22, U23, and U24 to form part of an S-turn and to form stacked base triples that constitute the heart of the theophylline binding site. This arrangement is in marked contrast to the ligand-free structure, where the binding pocket is collapsed and C27 is stacked between G26 and A28. Both theophylline and TAL2 form a base triple with C22 and U24, engaging both the 5- and 6-member rings of theophylline, but engaging only one of the 6-member rings of TAL2. The large conformational rearrangement of the RNA that occurs upon ligand binding is in good agreement with previous biochemical and computational models (21–23).

Divalent metal ions (Mg^2+^, Mn^2+^, or Co^2+^) are required for binding of theophylline to the aptamer (18, 27). An NMR study involving titration of paramagnetic Mn^2+^ into a Mg^2+^-containing complex suggested the presence of a specifically bound metal, which was modeled qualitatively as lying close to residues C22, U23, and U24 of the S-turn (27). The X-ray crystal structure of the aptamer–theophylline complex reveals a Mg^2+^ ion bound to N7 of G25 and coordinated to five water molecules, which stabilizes the S-turn that shapes the ligand binding site (Fig. 4*C*). Two Na^+^ ions assist in stabilizing this structure: the first coordinates to N7 of A5, O4 of U6, and the 2′-hydroxyl of C22; the second coordinates to both O2 and the 2′-hydroxyl of U23 and forms water-mediated hydrogen bonding interactions with the sugar-phosphate of U24 and the phosphate of A28.

The highest-affinity ligand that emerged from the screen is TAL1, which gave crystals that contain four complexes in the asymmetric unit, providing excellent electron density information for the bound ligand (*SI Appendix*, Fig. S5*C*). This compound binds in a similar manner to theophylline and TAL2 but forms two additional hydrogen bonds with the RNA. C22 is again engaged through N3 and the exocyclic amine, as well as a third interaction with O2. U24 is again engaged through N3, as well as a second interaction with O2. In order to accommodate TAL1 within the pocket, U24 is rotated outward by 0.7 Å compared to its orientation in the theophylline-bound complex.

TAL3 is chemically very similar to TAL2 and binds similarly to the aptamer (Fig. 4*E*). Thus, subtle differences must account for the 2.5-fold lower affinity of TAL3 compared to TAL2, which is mostly due to a difference in off-rate. TAL4 is more distinct from the other quinazolinones and has a 10–30-fold lower affinity for the aptamer, although still higher affinity than theophylline. The crystal structure reveals that TAL4 also binds in the theophylline pocket but forms only one hydrogen bond with the RNA, involving N3 of U24 (Fig. 4*F*). Nonetheless, the shape complementarity is excellent, allowing the formation of the critical stacking interactions. TAL4 is also unusual in that it contains a butanol–piperazine tail that is exposed to the solvent. The ability to tolerate this additional component demonstrates the substantial opportunity for chemical substitution of the core scaffold.

## Discussion

Given the prevalence of functional RNAs in biology, including their relevance to human disease, there has been a surge of interest in therapeutic interventions that target RNA. Approaches that are directed at the primary sequence of RNA, including antisense oligonucleotides, siRNAs, RNA editing, and synthetic mRNAs, have shown considerable promise. There also is the tantalizing possibility that disease-related RNAs can be targeted with orally bioavailable, low-molecular-weight compounds, which continue to make up the bulk of the modern pharmacopeia. For this promise to be realized, the formidable capabilities of contemporary small-molecule drug discovery, including high-throughput screening, structure-based design, and medicinal chemistry, all will need to be brought to bear on this less familiar class of targets.

The present study sought to investigate the extent to which these drug discovery capabilities can be applied to a tertiary structured RNA, using the well-studied theophylline aptamer as a test case. The theophylline ligand is itself a commonly used drug for the treatment of asthma and other pulmonary diseases. To obtain the theophylline aptamer, eight rounds of in vitro selection were carried out, starting from a pool of 10^14^ random-sequence RNA molecules (18). During the first five rounds, the selection was based on the ability of the RNA to bind theophylline, and during the last three rounds, a negative selection step was added to exclude molecules that also could bind caffeine. As a result, an aptamer was identified that is well adapted to binding theophylline with high affinity and selectivity, just as structured RNAs in biology have been evolved to meet fitness constraints relevant to the host organism. High-throughput screening can be regarded as a technological counterpart of biological evolution, with the power to discover compounds that meet various user-defined criteria. High-throughput screening is a single-harvest procedure, but through counter-screens and secondary screens, as well as multiple rounds of medicinal chemistry optimization, initial hits can be advanced toward compounds with more refined properties. The guidance provided by high-resolution structural data is often a key component of this hit maturation process.

Following the development of a suitable assay, high-throughput screening was used to identify ~11,000 hits that appear to bind the theophylline aptamer, of which 153 were validated through counter-screens and secondary assays. This level of attrition is typical of a high-throughput screening campaign. Theophylline and 3-methylxanthine were among the validated hits, but attention was focused on 46 hits that were more potent than theophylline in the strand displacement assay. These hits include four compounds that bind the aptamer with 2- to 340-fold greater affinity than theophylline, each occupying the same binding pocket as theophylline. X-ray crystallography studies revealed the structure of the aptamer in both the absence and presence of the ligands, demonstrating the large conformational change that occurs upon ligand binding. The structural data also revealed how three bound cations help to stabilize the binding pocket by coordinating to multiple residues of the RNA.

The set of crystal structures suggests an obvious path toward the improvement of compound potency, preserving the planar 6,6 heterocycle scaffold and the hydrogen-bonding interactions with nucleotides C22 and U24 and exploring alternative substitutions of the solvent-exposed portion of the ligand. TAL1 and the dimethoxy-quinazolinone core of the other three hits have a molecular weight of 193.1 and 206.2 Da, respectively, which is similar to that of theophylline (180.2 Da), thus demonstrating good ligand efficiency to achieve a K_d_ as low as 2.0 nM for binding to the RNA. There is little reason to pursue a medicinal chemistry campaign for compounds that lack a medicinal purpose, but it would be intriguing to evolve the aptamer further to determine whether it can be made to bind to the newly identified ligands even more tightly, seeking to understand the limits of RNA recognition of small molecules.

## Materials and Methods

### Synthesis of RNA.

The sequences of all oligonucleotides used in this study are listed in *SI Appendix*, Table S1. The RNAs were either purchased from Integrated DNA Technologies (Coralville, IA) or prepared by solid-phase synthesis using an Expedite 8909 DNA/RNA synthesizer, with reagents and phosphoramidites from either ChemGenes (Wilmington, MA) or Glen Research (Sterling, VA). Following synthesis, the RNAs were purified either by denaturing polyacrylamide gel electrophoresis and subsequent ethanol precipitation or by reverse-phase HPLC. The latter used a C18 column (Agilent, Santa Clara, CA) warmed at 65°C, with elution buffers of 0.1 M triethylammonium bicarbonate (pH 8.5) in either water or 50% acetonitrile and a gradient of 5–25% of the second buffer over 10 min at a flow rate of 30 mL/min. The collected eluate was evaporated to dryness in a vacuum centrifuge. Unless otherwise stated, all chemicals were from Sigma-Aldrich (St. Louis, MO).

### Strand Invasion Assay.

Assays were conducted in custom 1536-well, black, clear-bottom plates (#789076-A, Greiner Bio-One, Monroe, NC). The wells were prespotted with 5–50 nL compound in DMSO (5–50 µM final concentration) using an Echo 555 acoustic liquid handler (Beckman Coulter Life Sciences, Indianapolis, IN). To each well was added 2.5 µL 25 nM theophylline aptamer RNA, which was labeled at the 3′ end with Cy5 fluorophore (Fig. 1*A*). The mixture was incubated at room temperature for 10 min, and then, 2.5 µL 100 nM invading strand RNA was added; that RNA contained 2′-deoxy,2′-fluoronucleotides at all positions and labeled at the 5′ end with the BBQ-650 quencher. Both RNAs were provided in a mixture of 10 mM NaCl, 5 mM MgCl_2_, 20 mM HEPES (pH 7.5), and 0.01% Tween-20. The plate was briefly spun, incubated at 37°C for either 30 or 60 min, and then cooled to room temperature over 10 min. The wells were scanned for fluorescence using a PHERAstar FSX microplate reader (BMG LABTECH, Ortenberg, Germany), with filters for excitation and emission of 640 nm and 680 nm, respectively. For some assays, 2 mM spermidine and 2 µM tRNA were also present in the mixture. Ample time was provided for ligand binding to reach equilibrium for compounds that bind the aptamer with comparable on- and off-rates as theophylline. It should be noted, however, that the fluorescence assay was carried out under preequilibrium conditions with regard to strand invasion, given that the approach to equilibrium has a half-life of ~40 min under the assay conditions.

### High-Throughput Screening.

An initial screen was carried out with 1,107,541 compounds at 50 µM concentration, assaying their ability to inhibit strand invasion of the theophylline aptamer by a partially complementary oligonucleotide, which results in increased fluorescence (Fig. 1). The assay mixture contained 10 mM NaCl, 5 mM MgCl_2_, 20 mM HEPES (pH 7.5), and 0.01% Tween-20 at room temperature. Each of the 10,742 hits from the initial screen was then evaluated at both 5 and 30 µM concentration in four different screening formats. Format 1 was a repeat of the initial screen, measuring increased fluorescence. Format 2 was a repeat of the primary screen, with spermidine and tRNA added to the mixture to increase the stringency of the assay. Format 3 was a strand invasion assay with the theophylline aptamer labeled both at the 5′ end with Cy5 fluorophore and at the 3′ end with the IBRQ quencher, and an unlabeled invading strand was used so that compounds that inhibited strand invasion resulted in decreased fluorescence. Format 4 was a fluorescence polarization assay using a split form of the theophylline aptamer, consisting of 50 nM 5′-Cy3-labeled RNA having the sequence 5′-GAGCGAUACCAGCGAC-3′ and 75 nM unlabeled RNA having the sequence 5′-CGCCCUUGGCAGCGCUC-3′. Stabilization of the complex by a screened compound results in increased emission of polarized light due to reduced molecular rotation of the complex compared to the individual strands. The conditions for the four secondary screens were as above, except that formats 1, 3, and 4 contained 100 mM NaCl, 100 mM HEPES (pH 7.5), and 0.01% Triton-X100, and format 2 contained 200 mM NaCl, 100 mM HEPES (pH 7.5), 0.02% Triton-X100, 2 mM spermidine, and 2 µM tRNA.

### Surface Plasmon Resonance.

CM5 sensor chips (Cytiva Life Sciences, Marlborough, MA) were prepared by amine coupling of NeutrAvidin (ThermoFisher, Waltham, MA). The RNA aptamer was modified by inclusion of biotin and a (dA)_10_ spacer at the 3′ end. The running buffer contained 150 mM NaCl, 5 mM MgCl_2_, 10 mM HEPES (pH 7.4), and 0.01–0.1% DMSO. The RNA was present at 2 µg/mL concentration and was heated at 95°C for 2 min and then quickly cooled on ice. The ligand was added at a concentration of 0 to 0.1–2.0 µM (depending on affinity) and binding data were obtained using a Biacore 8K or T200 surface plasmon resonance system (Cytiva Life Sciences), run at 25°C at a flow rate of 30 µL/min.

### X-ray Crystallography.

Prior to crystallization of the aptamer–ligand complex, the RNA was resuspended at 0.4 mM concentration in 30 mM NaCl, 5 mM MgCl_2_, and 5 mM HEPES (pH 7.5) and refolded by heating at 95°C for 1 min and then cooling to room temperature over 20 min. The RNA was mixed with 1 mM ligand and then incubated at room temperature for 20 min. Crystals with theophylline, TAL2, TAL3, and TAL4 were grown at 4°C using the sitting-drop vapor diffusion method, with 40 nL aptamer–ligand solution and an equal volume of reservoir solution containing 0.1 M sodium cacodylate (pH 6.5), 5% (w/v) PEG 8K, and 39.5–41.0% (v/v) 2-methyl-2,4-pentanediol. The crystals were frozen in liquid nitrogen prior to data collection. Crystals of the aptamer–TAL1 complex were grown from 0.2 M Ca(OAc)_2_ and 20% (w/v) PEG 3350 at 20°C and were cryoprotected prior to freezing by diluting the reservoir solution with 100% glycerol to a final glycerol concentration of 20%, supplementing with 0.5 mM ligand. All data were collected at 0.97648 Å wavelength at ALS beamline 503, except for the aptamer–TAL4 complex, which were collected at SSRL beamline 9-2 at the same wavelength.

The X-ray diffraction data were processed with AutoProc. Atomic coordinates of the previously reported NMR structure of the theophylline aptamer (19) (PDB ID 1O15) were used as a search model for molecular replacement using REFINE within the PHENIX package. Structure refinement was carried out in PHENIX, alternating with manual fitting in Coot. Restraints for all the ligands were generated with eLBOW, integrated with the PHENIX package. The structures were solved in P1 symmetry, with two molecules in the asymmetric unit. Crystals of the aptamer–TAL1 complex contained four copies in the asymmetric unit (pairwise RMSD 0.18–0.42 Å), with minor differences in the structure of the GAAA tetraloop and around nucleotide C27. Ions were assigned based on geometry (28, 29).

For crystallization of the Fab–aptamer–ligand complex, the RNA-binding Fab (25) was transiently expressed in HEK293 cells, grown in 3 L volume, with second-day feed, and harvested at 6 L volume. The protein was purified using a HiTrap Protein G HP antibody purification column followed by a HiLoad Superdex 200 size exclusion column (Cytiva Life Sciences) and then concentrated to 16.8 mg/mL in phosphate-buffered saline (total yield 48.8 mg), which was stored at 80°C. Following gel purification, the RNA was dissolved in water, then refolded by heating at 95°C for 1 min, incubated at 50°C for 15 min in 30 mM KCl, 10 mM MgCl_2_, and 10 mM Tris-HCl (pH 7.4), cooled to room temperature over 5 min, and kept on ice for 5 min. Formation of the Fab–RNA complex was carried out as previously described (25), incubating the RNA with 1.1 equivalents of the Fab at room temperature for 30 min and then concentrating to 6 mg/mL using a 10-kDa cut-off Amicon Ultra-15 centrifugal device (Sigma-Aldrich).

Crystals of the Fab–aptamer complex were grown using the sitting-drop vapor diffusion method, with 40 nL Fab–aptamer solution and an equal volume of reservoir solution containing 16% (w/v) PEG 3350 and 0.1 M citrate bis-Tris propane (pH 8.8) at 20°C. The crystals were cryoprotected in 30% glycerol before freezing in liquid nitrogen. Data were collected at ALS beamline 503 and processed with AutoPROC. A molecular replacement solution was obtained using the Fab structure from PDB ID 6B14 as a search model, splitting chain H at residue 122 and chain L at residue 104. Modeling of the nucleotide portion of the structure was performed by manual fitting in Coot. To obtain the ligand-bound structure, Fab–aptamer crystals were soaked in a solution of 1.1 mM TAL2 for 1 h before freezing in 30% glycerol. Data were collected at ALS beamline 503 and processed with HKL2000 software. Nucleotides were modeled using the NMR structure of the aptamer as a guide (19). TAL2 could not be placed in the binding pocket other than by reference to the high-resolution cocrystal structure obtained in the absence of the Fab.

## Supplementary Material

Appendix 01 (PDF)

## Data Availability

All crystallographic data have been deposited in the Worldwide Protein Data Bank (http://wwpdb.org), with the following accession numbers: aptamer–Fab, 8D29; aptamer–Fab–TAL2, 8DK7; aptamer–theophylline, 8D28; aptamer–TAL1, 8D5L; aptamer–TAL2, 8D2B; aptamer–TAL3, 8D2A; and aptamer–TAL4, 8D5O.
