# Supplementary material for: Discovery of small molecules that target a tertiary-structured RNA
Source: Proc Natl Acad Sci U S A. 2022 Nov 21;119(48):e2213117119. doi: 10.1073/pnas.2213117119 (PMC9860313; doi:10.1073/pnas.2213117119)
Supplement: Supplementary file 1 — Appendix 01 (PDF) [file pnas.2213117119.sapp.pdf]

Supporting Information for

Discovery of Small Molecules that Target a Tertiary-Structured RNA

Elena Menichelli[1,2], Bianca J. Lam[1,3], Yu Wang[1], Vivian S. Wang[1], Jennifer Shaffer[1], Katrina F. Tjhung[1,4], Badry Bursulaya[1], Truc Ngoc Nguyen[1], Todd Vo[1], Phillip B. Alper[1], Christopher S. McAllister[1], David H. Jones[1], Glen Spraggon[1], Pierre-Yves Michellys[1,5], John Joslin[1], Gerald F. Joyce[1,6]*, and Jeff Rogers[1,7]*

[1]Novartis Institutes for BioMedical Research, San Diego, CA 92121; [2]Arrakis Therapeutics, Waltham, MA 02451; [3]Velia Therapeutics, San Diego, CA 92130; [4]Trotana Therapeutics, San Diego, CA 92121; [5]Odyssey Therapeutics, Cambridge, MA 02142; [6]The Salk Institute, La Jolla, CA 92037; [7]Radial Therapeutics, Cambridge, MA 02142

*To whom correspondence may be addressed. Email: gjoyce@salk.edu or jrogers@radialtx.com.

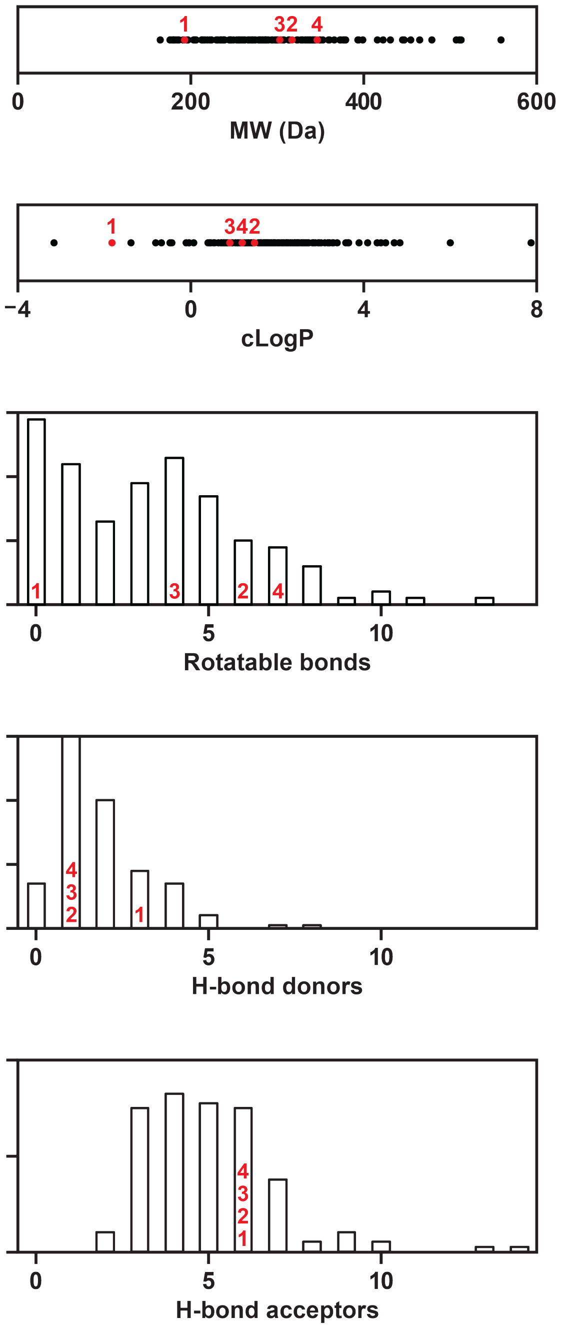

Fig. S1. Chemical properties of 153 confirmed screening hits. The distribution of values for molecular weight (MW), calculated partition coefficient between *n*-octanol and water (cLogP), number of rotatable bonds, and number of hydrogen-bond donors and acceptors are shown. Values for TAL1, TAL2, TAL3, and TAL4 are indicated by corresponding red numbers.

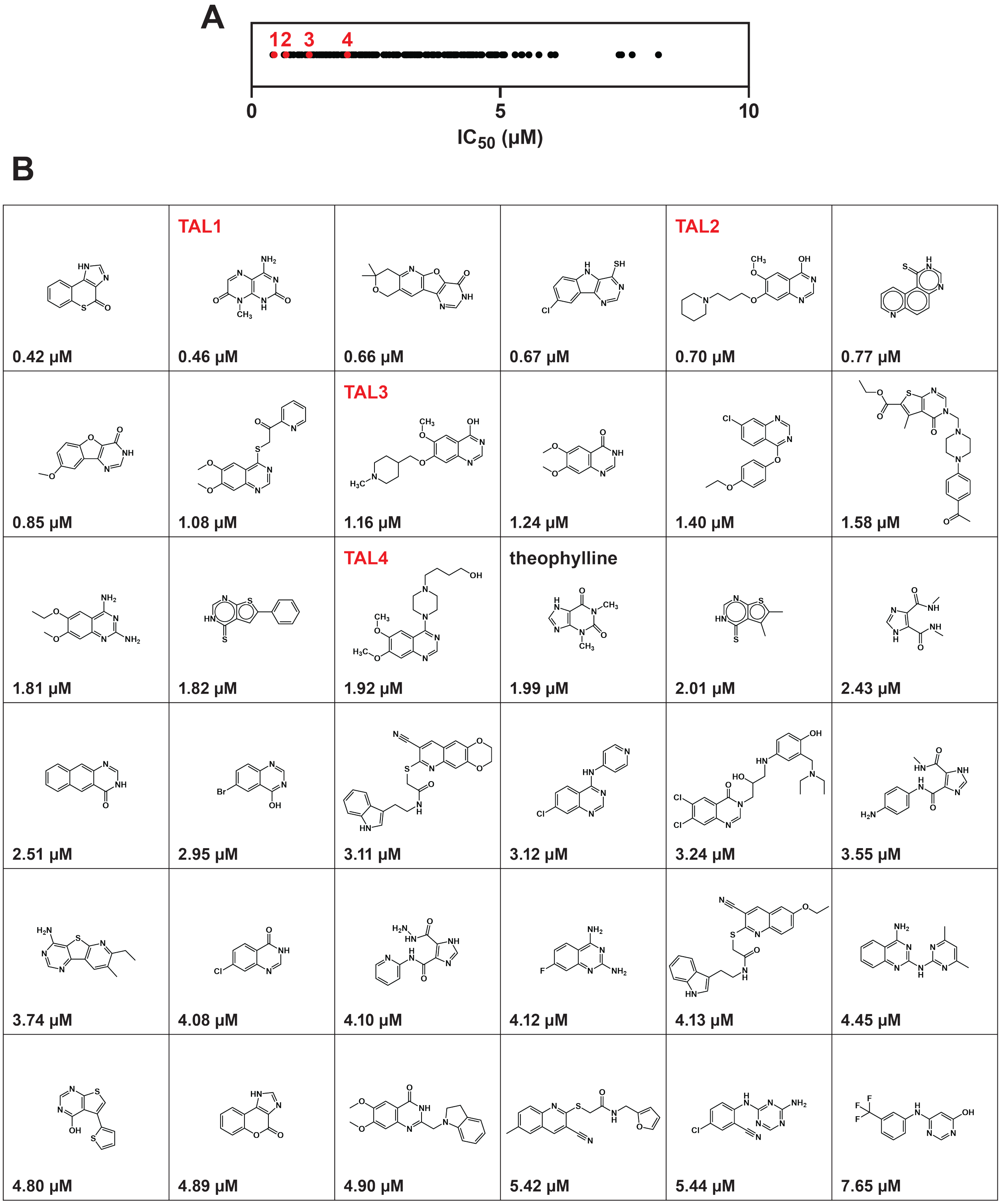

Fig. S2. Behavior of confirmed screening hits in strand invasion assay. (*A*) Distribution of IC_{50} values for all 153 confirmed hits, with values for TAL1, TAL2, TAL3, and TAL4 indicated by corresponding red numbers. (*B*) Chemical structure and IC_{50} values for theophylline and 35 representative hit compounds.

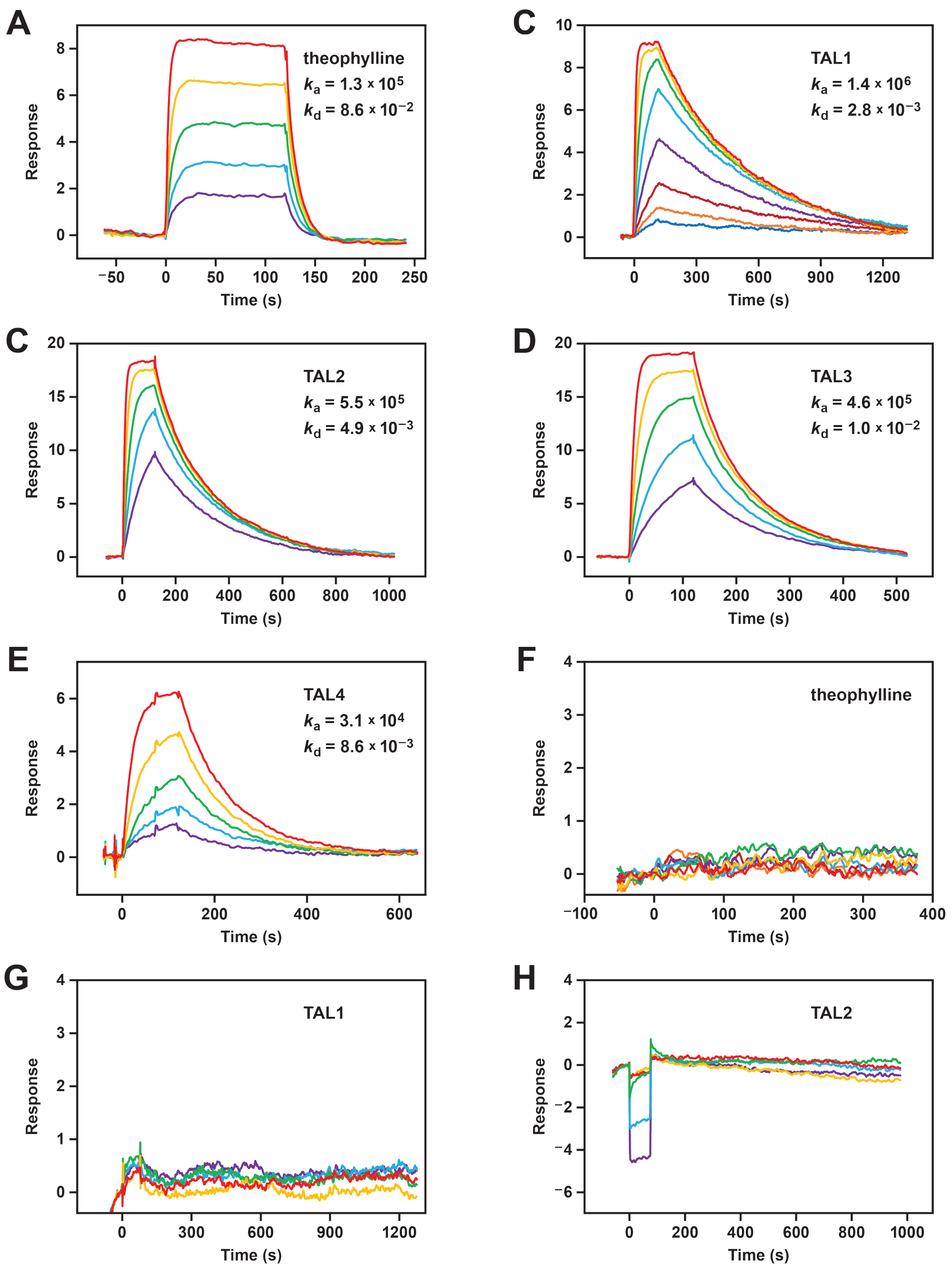

Fig. S3. Determination of ligand binding affinity by surface plasmon resonance. The on-rate (k_a, M^{-1} s^{-1}) and off-rate (k_d, s^{-1}) for binding of each compound to immobilized theophylline aptamer was determined for 0 to 0.1–2.0 μM ligand (depending on affinity), measured in the presence of 150 mM NaCl and 5 mM $MgCl_2$ at pH 7.4 and 25 °C. (*A*–*E*) interaction of theophylline and each of the four hit compounds with the canonical theophylline aptamer. (*F*–*H*) lack of interaction of theophylline, TAL1, and TAL2 with a mutant form of the theophylline aptamer that contains a U-to-C substitution at position 24.

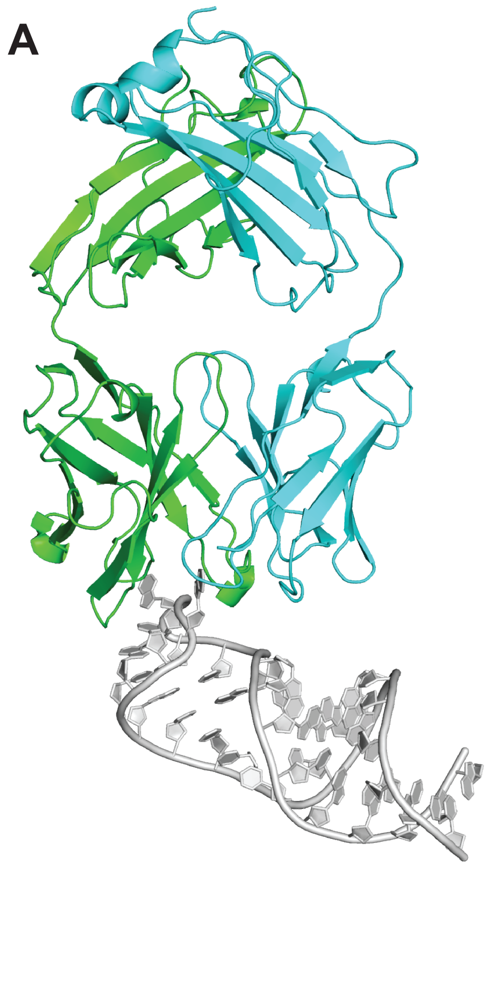

Fig. S4. Crystal structure of the theophylline aptamer in complex with a Fab that binds the distal hairpin loop of the RNA. (*A*) The ligand-free aptamer (gray) in complex with the Fab (heavy chain in green, light chain in cyan). (*B*) Soaking the ligand-free crystal with TAL2 converts the RNA to the ligand-bound conformation.

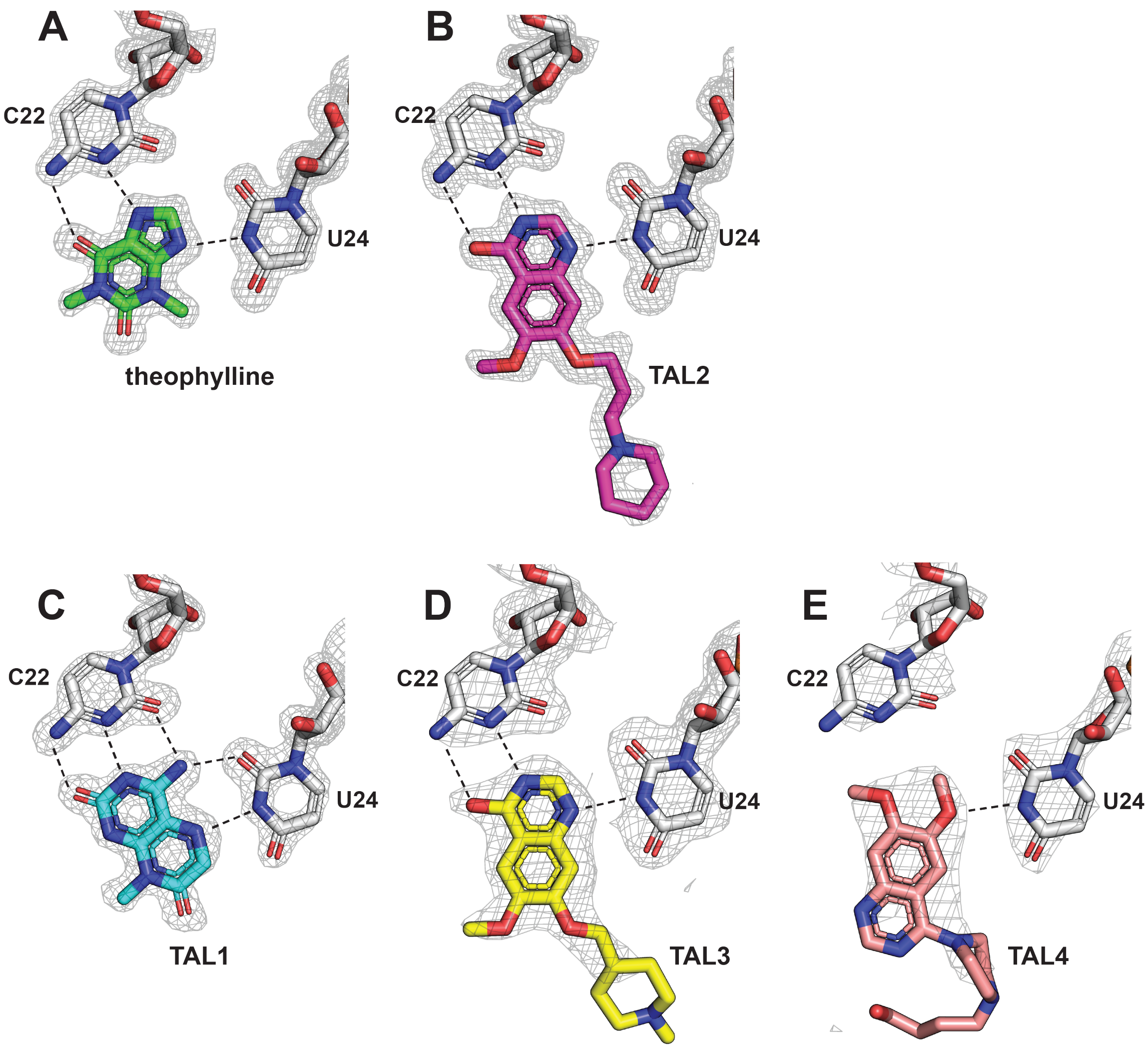

Fig. S5. Fo–Fc omit maps with the ligand, C22, and U24 omitted from the model to support placement of the ligand and paired nucleotides within the crystal structures. Omit maps are contoured at 2.5σ and shown as gray wire mesh. (*A*) theophylline, (*B*) TAL2, (*C*) TAL1, (*D*) TAL3, (*E*) TAL4. Regions with weak or missing density correspond to the solvent exposed tails of TAL2, TAL3, and TAL4.

Table S1. Sequences of oligonucleotides used in this study

Name	Source	Sequence (5´→3´)
aptamer-F	NVS	GGCGAUACCAGCCGAAAGGCCCUUGGCAGCGUC-Cy5
Q-invader	NVS	BBQ-2´-fluoro(GACGCUGCCAAGG)
F-aptamer-Q	IDT	Cy5-GGCGAUACCAGCCGAAAGGCCCUUGGCAGCGUC-IBRQ
invader	IDT	2´-fluoro(GACGCUGCCAAGG)
5´-split aptamer	IDT	Cy3-GAGCGAUACCAGCGAC
3´-split aptamer	IDT	CGCCCUUGGCAGCGCUC
aptamer-B	NVS	GGCGAUACCAGCCGAAAGGCCCUUGGCAGCGUC-d(A_{10})-biotin
mutant aptamer-B	NVS	GGCGAUACCAGCCGAAAGGCCCUCGGCAGCGUC-d(A_{10})-biotin
pentaloop aptamer	NVS	GGCGAUACCAGCGAAACACGCCCUUGGCAGCGUC
stabilized aptamer	NVS	GGCGAUACCAGCCGAAAGGCCCUUGGCAGCGCC

The molecules were either synthesized in-house (NVS) or purchased from IDT. Nucleotides in red are changed relative to the canonical theophylline aptamer. Cy5, cyanine 5 fluorophore; BBQ, BlackBerry BBQ-650 quencher; IBRQ, Iowa Black RQ quencher; Cy3, cyanine 3 fluorophore.

Table S2. X-ray crystallography data collection and refinement statistics

	Ligand-free Fab	TAL2-soaked Fab	Theophylline	TAL1	TAL2	TAL3	TAL4
**Data collection**							
Space group	P1	P1	P1	P1	P1	P1	P1
Cell dimensions							
a, b, c (Å)	76.15, 92.85, 93.64	38.39, 90.12, 93.75	23.07, 29.56, 80.40	25.60, 50.27, 79.85	23.07, 29.57, 80.31	23.08, 29.66, 80.29	22.97, 29.39, 80.31
α, β, γ (°)	97.35, 90.32, 106.92	83.53, 89.40, 84.57	95.73, 90.09, 109.58	94.28, 91.53, 90.13	95.66, 91.51, 109.96	95.39, 89.99, 109.00	84.67, 90.03, 70.44
Resolution (Å)	68.66–1.81 (1.87–1.81)	89.14–2.46 (2.55–2.46)	39.97–1.42 (1.47–1.42)	41.06–1.64 (1.70–1.64)	20.09–1.44 (1.49–1.44)	27.91–2.17 (2.25–2.17)	39.96–2.70 (2.78–2.70)
R_{merge}	0.1327 (0.8311)	0.0497 (0.4341)	0.0266 (0.3014)	0.1318 (0.4013)	0.0705 (0.4668)	0.0536 (0.3862)	0.0439 (0.0896)
$I/\sigma I$	7.92 (2.82)	15.53 (2.79)	15.19 (2.51)	8.30 (2.33)	11.26 (2.41)	8.78 (2.27)	18.13 (11.02)
Completeness (%)	93.74 (93.93)	92.06 (96.35)	93.76 (91.54)	95.87 (93.84)	93.35 (72.72)	91.52 (84.90)	82.52 (66.85)
Redundancy	3.8 (2.9)	3.7 (3.2)	2.1 (2.1)	3.2 (2.3)	3.8 (3.6)	2.0 (2.0)	3.3 (2.8)
**Refinement**							
Resolution (Å)	68.66–1.81	89.14–2.46	39.97–1.42	41.06–1.64	20.09–1.44	27.91–2.17	39.96–2.70
No. reflections	207765 (20804)	41477 (4384)	35204 (3450)	46388 (4612)	33417 (2581)	9718 (911)	4498 (365)
R_{work}/R_{free}	0.176/0.211	0.246/0.270	0.174/0.208	0.193/0.221	0.200/0.225	0.187/0.252	0.238/0.271
No. non-H atoms							
Protein	13181	6563	–	–	–	–	–
RNA	2892	1446	1408	2816	1408	1408	1408
Ligand/ion	6	–	35	71	55	50	50
Water	2043	43	362	545	319	124	–
B-factors							
Protein	24.0	77.3	–	–	–	–	–
RNA	102.3	152.8	22.9	26.3	18.5	37.9	24.1
Ligand/ion	24.7	–	17.0	17.7	19.1	33.2	19.9
Water	34.5	49.8	31.0	28.5	25.0	29.0	–
R.m.s. deviation							
Bond lengths (Å)	0.009	0.024	0.006	0.006	0.007	0.006	0.015
Bond angles (°)	1.21	1.10	1.06	1.07	1.12	1.07	1.97

Values in parentheses are for the highest-resolution shell. Two crystals were used for the TAL2-soaked Fab dataset; one crystal was used for each of the other datasets.
